# Resistance to noise-induced gap detection impairment in FVB mice is correlated with reduced neuroinflammatory response and parvalbumin-positive neuron loss

**DOI:** 10.1038/s41598-020-75714-1

**Published:** 2020-11-24

**Authors:** Alexander K. Zinsmaier, Weihua Wang, Li Zhang, Nadia N. Hossainy, Shaowen Bao

**Affiliations:** Department of Physiology, University of Arizona College of Medicine, Tucson, AZ 85724 USA

**Keywords:** Sensory processing, Cortex

## Abstract

Exposure to loud noises results in neuroinflammatory responses in the central auditory pathway. Noise-induced neuroinflammation is implicated in auditory processing deficits such as impairment in gap detection. In this study, we examined whether strain differences between the FVB and C57BL/6 mice in noise-induced impairment in gap detection are correlated with strain differences in neuroinflammatory responses. We found that noise induced more robust TNF-α expression in C57BL/6 than in FVB mice. Noise-induced microglial deramification was observed in C57BL/6 mice, but not in FVB mice. Furthermore, noise exposure resulted in a reduction in parvalbumin-positive (PV+) neuron density in the C57BL/6 mice, but not in FVB mice. These results suggest that neuroinflammatory responses and loss of PV+ neurons may contribute to strain differences in noise-induced impairment in gap detection.

## Introduction

Hearing loss often results in impairment in gap detection^1^, which is considered a sign of temporal processing deficit and possibly tinnitus^2,3^. In animal models, gap detection is often examined with the gap prepulse-induced inhibition of the startle response^2^. Impairment in this behavioral paradigm was correlated with several pathological changes in the auditory pathway, such as enhanced excitatory synapses, weakened inhibitory synapses and increased neuronal membrane excitability^4–13^. A potential modulator of these synaptic and membrane properties is the neuroinflammatory process and the proinflammatory cytokine TNF-α^14–18^. Hearing loss causes inflammatory responses along the central auditory pathway, and an increase in the expression of several proinflammatory cytokines^19–22^. Among them, TNF-α has been shown to play a central role in organizing the inflammatory response in the brain. Its expression is rapidly upregulated following noise-induced hearing loss in mice, followed by microglial activation and increased expression of other proinflammatory cytokines^22^. Blocking noise-induced increase of TNF-α expression prevented both synaptic imbalance and behavioral impairment in gap detection in noise exposed mice^22^. In the same study, blocking TNF-α expression also prevented behavioral evidence of tinnitus assessed with an operant perceptual task^22^. In humans, polymorphisms of several inflammatory cytokines, including TNF-α, are associated with the risk of noise-related tinnitus^23–25^, a central auditory disorder that is often comorbid with impairment in gap detection^3,26^. The level of interleukin 10, an anti-inflammatory cytokine also known as human cytokine synthesis inhibitory factor, was reduced in people with tinnitus, but not in people who had hearing loss without tinnitus^27^.

Strain differences in immune and neuroinflammatory responses have been widely reported in common mouse strains^28–30^. For example, the C57BL/6 and the FVB strains exhibit different immune response profiles, with C57BL/6 mice being Type 1 T helper cell-dominant (Th1-dominant), and FVB mice being Th2-dominant^31^. Th1 cells promote the secretion of proinflammatory cytokines, and Th2 cells promote the secretion of anti-inflammatory cytokines^32^. When challenged with experimentally induced stroke and reperfusion the two strains showed different profiles of immune cells activation^33^. In addition, C57BL/6 mice displayed greater neurological and motor deficits from the stroke than FVB mice^33^. Interestingly, strain differences were also reported between C57BL/6 and FVB mice in how noise exposure influenced inhibitory synapses and gap detection behavior^34^. Exposure to loud noises resulted in impaired gap detection in C57BL/6 but not FVB. The behavioral impairment is correlated with reduced expression of glutamic acid decarboxylase 65 (GAD65), an enzyme that produces the inhibitory transmitter GABA, also in C57BL/6 but not FVB mice^34^. The parallel between noise-induced impairments in gap detection and the proinflammation bias in C57BL/6 mice compared to the FVB mice raises the question whether there are strain differences in noise-induced neuroinflammatory responses that could account for the behavioral difference.

The strain difference between C57BL/6 and FVB mice in noise-induced impairment in gap detection was correlated with the reduction of GAD65 expression^34^. Many studies have implicated inhibitory synapses and inhibitory neurons in the gap detection. Weakened inhibitory synapses and reduced expression of glutamate decarboxylase in the central auditory pathway were reported in animal models of impaired gap detection^11,13,22,34^. Investigation of different types of inhibitory neurons further suggested specific involvement of PV+ neurons in gap detection. Cortical PV+ neurons can encode brief gaps, and artificially altering activity of those PV+ neurons influences gap detection performance^35,36^. Taken together, these findings raise the question whether noise exposure differentially impacts PV+ neurons in C57BL/6 and FVB mice.

In this study, we examined noise-induced neuroinflammatory responses in FVB and C57BL/6 mice, and determined whether PV+ neurons are differently impacted in the two strains of mice by the noise exposure. Our results indicate that noise-induced increase in TNF-α expression was significantly weaker in the FVB than C57BL/6 mice. Significant microglial deramification was observed in the C57BL/6 but not FVB mice. Noise exposure resulted in a significant reduction in cortical PV+ neuron density in C57BL/6 but not in FVB mice. These differences may potentially contribute to the strain difference in noise-induced impairment in gap detection.

## Results

### FVB mice do not show noise-induced impairment in gap detection

**Figure 1:**
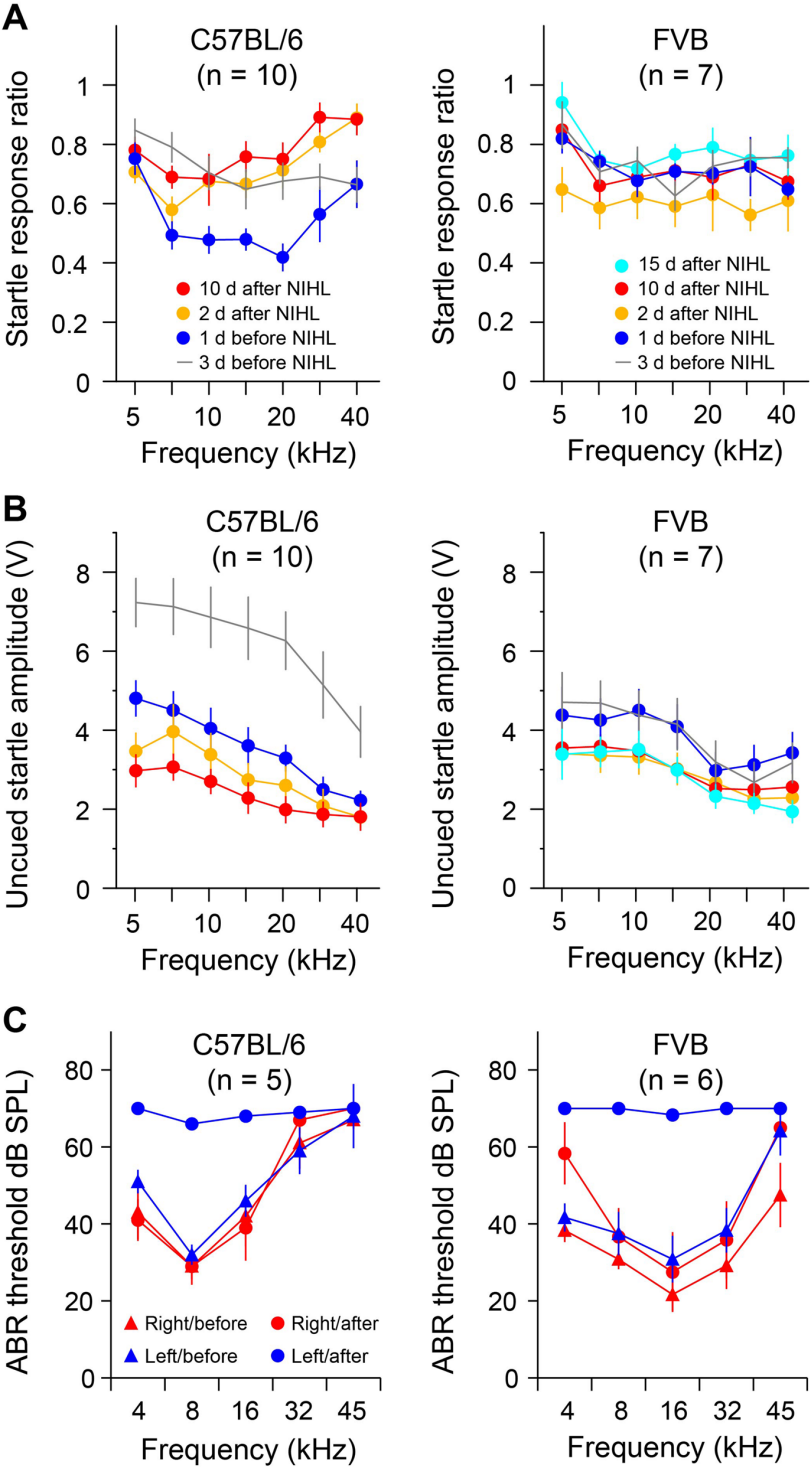
Strain differences in noise-induced impairment in gap detection. (**A**) Noise exposure resulted in impaired gap detection in C57BL/6 mice in a broad frequency range 2 and 10 days after the exposure. The same noise exposure did not significantly alter gap detection performances in FVB mice 2, 10 and 15 days after the exposure. (**B**) Noise exposure led to a significant reduction in startle response amplitude in C57BL/6 but not FVB mice. (**C**) Noise exposure produced similar levels of ABR threshold increases in C57BL/6 and FVB mice.

We first examined gap detection before and after noise exposure in two strains of mice—the C57BL/6 and the FVB. Ten C57BL/6 mice underwent gap detection tests in three phases: 3 daily sessions before, 2 days after and 10 days after the noise exposure (Fig. 1A). A frequency-by-session repeated measures ANOVA revealed significant effects of test sessions (*F*_3,27_ = 8.254, *p* < 0.001). Pairwise comparison indicated significant performance improvement over the three sessions before noise exposure (pairwise comparison, 3 days before vs. 1 day before, *p* = 0.015). Noise exposure significantly impaired gap detection compared to the final day before noise exposure (1 day before vs. 2 days after, *p* = 0.002; 1 day before vs. 10 days after, *p* < 0.001; 2 days after vs. 10 days after, *p* = 0.031; 3 days before vs. 2 days after, *p* = 0.973; 3 days before vs. 10 days after *p* = 0.350). Seven FVB mice underwent gap detection tests in four phases: 3 daily sessions before, 2 days, 10 days and 15 days after the noise exposure. Interestingly, we found no significant differences in gap detection performances across the test sessions (frequency-by-session repeated measures ANOVA, effect of sessions, *F*_4,24_ = 2.361, *p* = 0.082; frequency-by-session interaction *F*_24,144_ = 0.754, *p* = 0.787; Fig. 1A).

Previous reports have shown that noise exposure can lead to weakened startle responses^34^. In this study, we measured the startle response amplitude as the peak-to-trough amplitude of the piezoelectric sensor signal. The response amplitudes in uncued trials were plotted as a function of the frequencies of the background tones (Fig. 1B). In C57BL/6 mice, the startle response amplitude was reduced during course of the behavioral tests (frequency-by-session repeated measures ANOVA, effect of sessions, *F*_3,27_ = 19.401 and *p* < 0.0001; frequency-by-session interaction *F*_18,162_ = 4.253 and *p* < 0.0001; Fig. 1B). Pairwise comparisons indicated that there was a rapid reduction of startle amplitude during the first 3 days of tests before noise exposure (3 days before vs. 1 day before, *p* = 0.006), and subsequent noise exposure further suppressed the startle response amplitude (1 day before vs. 10 days after, *p* = 0.048). By contrast, we did not observe significant differences in the startle response amplitude in the FVB mice (frequency-by-session repeated measures ANOVA, effect of sessions, *F*_4,24_ = 1.758 and *p* = 0.170; frequency-by-session interaction *F*_24,144_ = 1.515 and *p* = 0.071; Fig. 1B). These results indicate that the gap detection performance was not correlated with the startle response amplitude^34^.

ABR thresholds were measured in a subset of the mice before and 15 days after noise exposure. Noise exposure resulted in up to 40 dB threshold shifts in both C57BL/6 and FVB mice (Fig. 1C). An exposure-by-side-by-frequency repeated measures ANOVA showed significant effects of noise exposure (*F*_1,4_ = 21.453 and *p* = 0.010 for C57BL/6, and *F*_1,5_ = 35.342 and *p* = 0.002 for FVB), and binaural differences (*F*_1,4_ = 13.669 and *p* = 0.021 for C57BL/6, and *F*_1,5_ = 19.931 and *p* = 0.007 for FVB). Pairwise comparisons indicated that significant threshold increases were observed only in the left ear after noise exposure for both C57BL/6 and FVB mice.

These results replicated previous findings of strain differences in noise-induced impairment in gap detection^34^, and extended previous findings to a wider frequency range (from 20 to 40 kHz). We also addressed the possibility that gap detection impairments were simply delayed in FVB mice by carrying out an additional test 15 days after the noise exposure.

### Noise exposure leads to neuroinflammatory responses in C57BL/6 but not FVB mice

**Figure 2:**
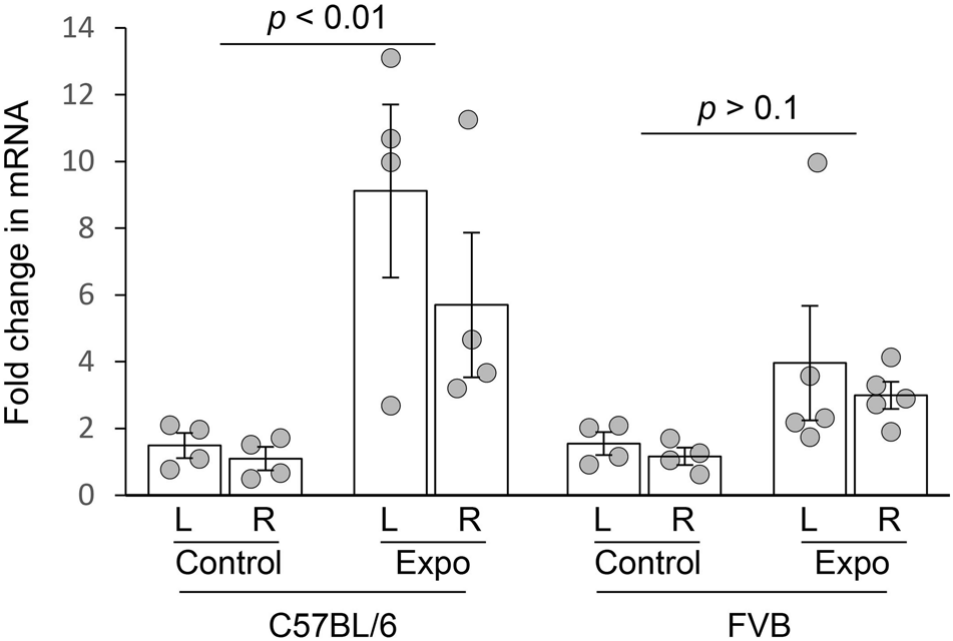
Strain differences in noise-induced increase of TNF-α expression. TNF mRNA level significantly increased in C57BL/6 but not FVB mice 10 days after noise exposure.

To determine if noise-induced neuroinflammatory responses are different between FVB and C57BL/6 mice, we measured the mRNA levels of the proinflammatory cytokine TNF-α. A strain-by-noise exposure-by-side repeated measures ANOVA showed a significant effect of noise exposure on TNF-α expression (*F*_1,12_ = 19.299, *p* < 0.001; Fig. 2). A post-hoc analysis indicated that noise resulted in a significant increase in TNF-α mRNA level in the C57BL/6 mice (*p* < 0.01) but not in the FVB mice (*p* > 0.1; Fig. 2).

**Figure 3:**
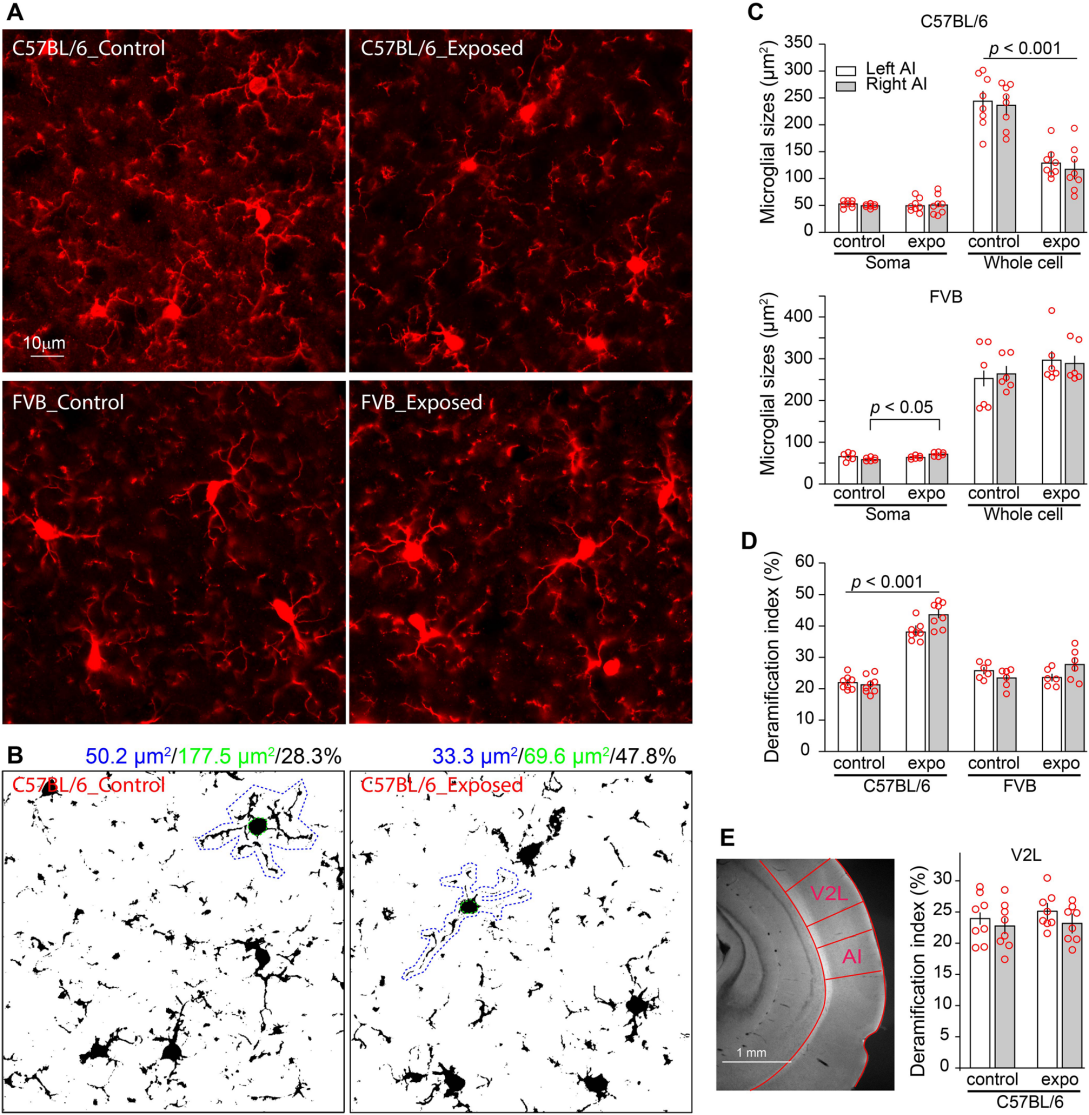
Strain differences in noise-induced microglial activation. (**A**) Example images of microglia in AI of the right hemisphere stained with IBA-1 antibody. (**B**) Microglial activation was assessed by morphological deramification index, which is the ratio of two-dimensional size of the soma and the size of whole microglial body including the processes. Shown here are binary images of the microglia. The whole bodies were outlined in blue and the somas were outlined in green. The soma and whole-body sizes and their ratios were given in blue, green and black colors. (**C**) Microglial soma and whole-body sizes. Noise exposure resulted in a significant reduction of the whole-body size in the C57BL/6 (n = 8) but not FVB (n = 6) mice. The soma size was not altered by noise exposure in C57BL/6 mice, and was increased in the right AI of FVB mice. (**D**) Deramification was observed 5 days after noise exposure in C57BL/6 but not FVB mice. (**E**) Noise-induced microglial deramification was not observed in the lateral area of the secondary visual cortex (V2L, n = 8), which is located dorsal to AI^40^.

We also assessed microglial deramification—the reduction of the size of dendrites relative to the size of the soma size (Fig. 3A,B)—as a measure of microglial activation^37–39^. A total of 48 sections of AI tissue were stained for IBA-1 and analyzed (2 sections/mouse, n = 4 mice for C57BL/6 or n = 3 mice for FVB). Noise exposure resulted in a significant reduction in whole-body size of the microglia in C57BL/6 but not FVB mice (noise exposure-by-side two-way ANOVA, effect of noise exposure, *F*_1,28_ = 66.645 and *p* < 0.001 for C57BL/6, and *F*_1,20_ = 0.193 and *p* = 0.665 for FVB; Fig. 3C). The size of the soma was not significantly altered by noise exposure in C57BL/6 mice (*F*_1,28_ = 0.050 and *p* = 0.825). There was a small but significant increase in the soma size in FVB mice after noise exposure and the difference was observed only for the right hemisphere (*F*_1,20_ = 4.999 and *p* = 0.037; pairwise comparison, *p* < 0.05 for the right side between control and noise-exposed).

We then used the soma-to-whole body size ratio as an index for microglial deramification. Noise exposure-induced microglial deramification was significantly different between the two strains (strain-by-noise exposure-by-side ANOVA, effect of noise exposure *F*_1,48_ = 74.758, *p* < 0.001; strain difference, *F*_1,48_ = 41,584, *p* < 0.001; strain-by-noise exposure interaction, *F*_1,48_ = 90.324, *p* < 0.001; Fig. 3D). A Post-hoc analysis showed that noise-induced deramification was significant for C57BL/6 mice but not FVB mice (*p* < 0.001 for C57BL/6 and *p* > 0.5 for FVB mice; Fig. 3D).

To determine whether noise-induced neuroinflammatory responses are localized to the auditory pathway, we analyzed the microglia in the lateral secondary visual cortex (V2L), which is located dorsal to AI on the same brain sections (Fig. 3E). Noise exposure did not lead to microglial deramification in V2L (noise exposure-by-side two-way ANOVA, effect of noise exposure, effect of noise exposure *F*_1,28_ = 0.459, *p* = 0.504; side difference, *F*_1,28_ = 1.826, *p* = 0.187; side-by-noise exposure interaction, *F*_1,28_ = 0.102, *p* = 0.752; Fig. 3E). These results are consistent with previous observation that noise exposure causes neuroinflammatory responses in the auditory cortex but not in the visual cortex^22^.

### Noise exposure causes PV+ neuron loss in C57BL/6 but not FVB mice

**Figure 4:**
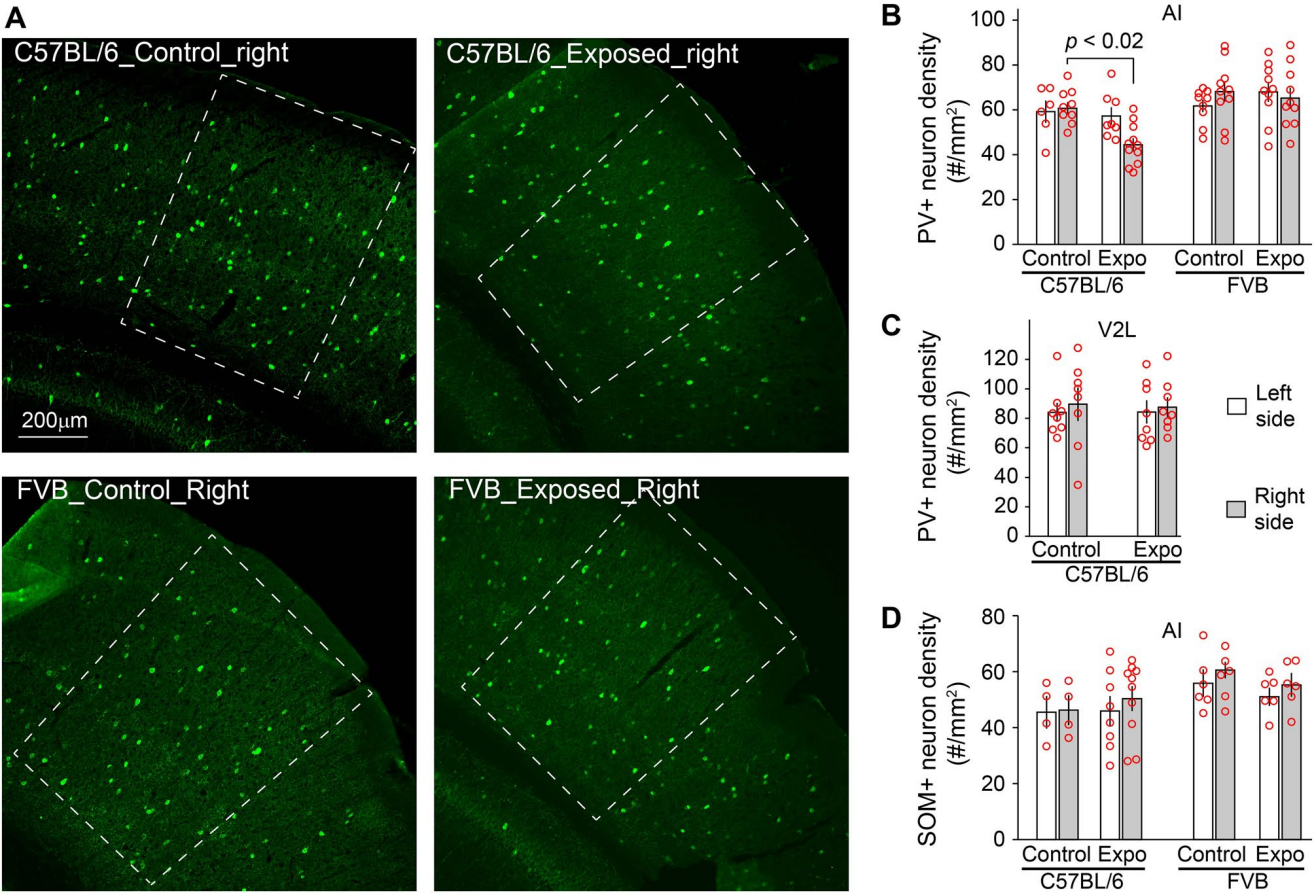
Strain differences in noise-induced PV+ neuron loss. (**A**) Example images of PV+ neurons in AI of the right hemisphere, which is delimited with white dashed lines. (**B**) The density of PV+ neurons was significantly reduced in the right AI of C57BL/6 but not FVB mice 5 days after noise exposure. (**C**) The reduction of PV+ neuron density was not observed in lateral area of the secondary visual cortex (V2L). (**D**) The density of SOM+ neurons was not significantly altered in AI of C57BL/6 or FVB mice 5 days after noise exposure.

Since PV+ neurons are implicated in representation and detection of gap in sound^35,36^, we investigated whether noise exposure affect PV+ neuron density in C57BL/6 and FVB mice. A total of 73 sections of AI tissue were stained for PV and analyzed (3–4 sections/mouse, 3 mice/group). We found a significant reduction of PV+ neuron density in AI of the right hemisphere (noise exposure-by-side ANOVA, effect of noise exposure, *F*_1,30_ = 8.492, *p* = 0.007; noise exposure-by-side interaction, *F*_1,30_ = 5.379, *p* = 0.027; Fig. 4A,B). The reduction of PV+ neuron density was not observed in the lateral area of the secondary visual cortex (V2L), which is located dorsal to AI^40^ (n = 8 for all groups. noise exposure-by-side ANOVA, effect of noise exposure, *F*_1,28_ = 0.018, *p* = 0.895; Fig. 4C). Interestingly, this noise-induced PV+ neuron loss was not observed in FVB mice (noise exposure-by-side ANOVA, effect of noise exposure, *F*_1,35_ = 0.175, *p* = 0.678; noise exposure-by-side interaction, *F*_1,30_ = 1.339 *p* = 0.255; Fig. 4B). Furthermore, the density of somatostatin + neurons were not altered in AI of either C57BL/6 (n = 4 for control left, n = 4 for control right, n = 8 for noise-exposed left, n = 10 for noise-exposed right; noise exposure-by-side ANOVA, effects of noise exposure, *F*_1,22_ = 0.182, *p* = 0.674; noise-by-side interaction, *F*_1,22_ = 0.111, *p* = 0.742; Fig. 4D) or FVB mice (n = 6 for all groups; effects of noise exposure, *F*_1,20_ = 0.694, *p* = 0.415; noise-by-side interaction, *F*_1,24_ = 0.018, *p* = 0.895; Fig. 4D). These results indicate that noise exposure results in a specific reduction in PV+ neuron density in the auditory pathway in C57BL/6 mice, but not in FVB mice. Whether the observed PV+ neuron loss is due to a decrease in PV expression or cell death has yet to be determined.

## Discussion

In this study, we showed that noise exposure resulted in neuroinflammatory responses and a reduction in PV+ neuron density in C57BL/6 mice but not in FVB mice. These strain differences are correlated with previously reported differences in noise-induced impairment in gap detection performance^34^, which was confirmed in this study.

Gap detection, as measured with gap prepulse-induced inhibition of the startle response, has been used to study temporal sound processing and tinnitus in mouse models^2,35^. The reported effects of noise exposure to gap detection are often variable^41^. As the published studies used different strains of mice, it is likely that strain differences contributed to the reported variability^13,34,42,43^. Investigating these strain differences could provide information about mechanisms underlying gap detection and noise-induced auditory processing deficits. In this study, we showed that noise-induced neuroinflammatory responses are different between C57BL/6 and FVB mice. Our findings support previously reported differences in brain immune response profiles and functional outcomes between the two strains after experimentally induced stroke and reperfusion^33^. Our results suggest that strain differences in neuroinflammatory response may contribute to the observed variability in gap detection following noise exposure.

Impaired gap detection has been observed in human tinnitus patients^3,26^, and is used to evaluate tinnitus in animal models^2,11,22,34,44^. Thus, the strain difference in noise-induced impairment in gap detection may represent a difference in susceptibility to noise induced tinnitus^34^, a potentially useful model to study the interaction between genes and experience in tinnitus etiology. Noise exposure and hearing loss are risk factors for tinnitus. However, not all people with hearing loss or a history of noise exposure develop tinnitus. Our results suggest that predisposition for neuroinflammatory responses may be a genetic risk factor for tinnitus. This interpretation is consistent with findings that polymorphisms of inflammatory cytokines, including TNF-α, are associated with the risk of tinnitus, especially in those with a history of occupational noise exposure^23–25^.

A potential link between neuroinflammation and impaired gap detection is reduced inhibitory neuron function. For example, weakened inhibitory synaptic transmission and reduced GAD65 expression were correlated with impairments in gap detection^11,13,22,34^, and blocking neuroinflammation prevented noise-induced reduction in inhibitory synaptic transmission^22^. In this study, we found strain differences in noise-induced neuroinflammatory responses in C57BL/6 mice are correlated with strain differences in the reduction in PV+ neuron density, which is consistent with the reported strain difference in noise-induced GAD65 reduction between C57BL/6 and FVB mice^34^. However, while the noise induced neuroinflammatory responses were bilateral as reported before^22^, the PV+ neuron loss in AI was limited to the right hemisphere. It is possible that, in addition to neuroinflammation, persistent hearing loss is also required for noise-induced PV+ neuron loss. Future research is needed to determine whether there are causal relationships between neuroinflammation, hearing loss and PV+ neuron loss, and between PV+ neuron loss and impairment in gap detection.

## Methods

### Animals and noise exposure

All procedures were approved by the University of Arizona IACUCs. All methods were performed in accordance with the NIH guidelines and Public Health Services regulations for the Humane Care and use of laboratory animals. C57 BL/6 J and FVB.129P2-Pde6b + Tyrc-ch/AntJ between 8 and 12 weeks of age were used (Jackson Laboratory, Bar Harbor, ME). Mice underwent either noise exposure or sham exposure using a procedure identical to that reported before^22,34^. Briefly, Animals were anesthetized with ketamine (100 mg/kg, i.p.) and xylazine (10 mg/kg, i.p.), and maintained at 36.5 °C with a homeothermic heating pad (Harvard Apparatus, Holliston, MA). Unilateral NIHL was induced by playing a continuous 112–114 dB SPL noise centered at 8 kHz through a custom-made piezoelectric earphone speaker to the left ear for 2 h. The right ear was protected with sound attenuating clay. The sound level was measured with a Bruel and Kjaer 4135 condenser microphone (Nærum, Denmark). This procedure has been shown to produce similar levels of hearing loss in exposed ear in C57BL/6 and FVB mice^34^. Sham-exposed controls underwent the same procedure except that noise was omitted.

### Measurement of ABR threshold

Hearing thresholds were assessed under anesthesia using ABR immediately before and after (5, 15 and 20 days) the noise exposure procedure. ABR signals were recorded using the BioSigRP software on a TDT RX5 Sys3 recording rig. Tone pips (3 ms full-cycle sine waves at 4, 8, 16, 32 and 45 kHz at 5 dB intensity steps from 0 to 70 dB) were delivered to the left ear through a cannulated speaker at a rate of 19 times per second. The speaker was calibrated to have < 3% harmonic distortion and a flat output in the entire frequency range (Tucker-Davis Technologies SigCal32). 500 recordings were averaged for each frequency intensity pair. ABR signals were recorded with electrodes subcutaneously inserted at three locations: behind the ear coupled with the speaker, at the vertex of the head, and near the base of the tail. ABR threshold was defined as the sound level at which one or more peaks were distinguishable by eye against the background activity.

### Behavioral test for gap detection

The procedure was modified from those of previous reports^22,34^. During the testing session, mice were placed in a plastic container with a mesh lid. The container was placed on a piezoelectric sensor in a sound attenuation chamber. Sounds were played through an open field speaker (FOSTEX FT17H) fixed above the container. The gap detection task measures the acoustic startle response elicited by a brief white noise pulse and its suppression by a preceding silent gap embedded in a continuous background sound. Each trial started with a carrier pure tone (frequency pseudorandomly selected from 5, 7, 10, 14, 20, 28 and 40 kHz, all at 75 dB SPL), played for a duration of 10–20 s. In uncued trials, the carrier tone was followed by a startle stimulus—a 50 ms white noise burst at 102 dB SPL. In cued trials, a 50-ms silent gap in the background sound was introduced starting 100 ms before the onset of the loud noise burst. In each testing session, the animal underwent a total of 500 trials (50% cued and 50% uncued). After each session, we calculated the startle response ratio, which is defined as the average startle amplitude in the silent gap-cued trials divided by the average amplitude in the uncued trials. A lower startle response ratio indicates better detection of the silent gap. A startle response ratio of 1 suggests that the animal failed to detect the silent gap. All mice started with 3 days of gap detection test to stabilize their performance. Their performance on the third day was used as the baseline for their gap detection behavior. On the fourth day, all animals underwent noise exposure as described above. Two days and 10 days after the noise exposure, gap detection was tested again, and the performances were compared with the baseline performance. FVB mice were given an additional test 15 days after noise exposure.

### Immunostaining of microglia, PV+ and SOM+ interneurons

The procedure was modified from those of previous reports^22,45^. Mice were transcardially perfused 5 days after noise-induced hearing loss under deep anesthesia with ice-cold PBS followed by 4% paraformaldehyde. Brains were removed and fixed in the same fixative overnight at 4 °C, equilibrated in 30% sucrose and embedded in Tissue-Tek (Sankura Finetek). Frozen coronal Sections (16 μm) were collected on gelatinized glass slides. After air drying, sections were washed in PBS and penetrated with 0.1% Triton-X at room temperature for 10 min. The samples were blocked with Dako Serum-free blocking buffer (Dako) and incubated with primary antibodies for ionized calcium binding adaptor molecule 1 (IBA-1 rabbit monoclonal, ab178846; Abcam), PV (PV rabbit polyclonal antibody, PA1-933, Invitrogen), or SOM (SOM mouse monoclonal antibody, sc-55565, Santa Cruz Biotechnology) overnight at 4 °C. Samples were then washed in PBS and incubated with the secondary antibody for IBA-1 or PV, conjugated with Alexa Fluor 568 (Invitrogen) or Alexa Fluor 488 (Invitrogen), 1 h at room temperature to enable fluorescent detection. After rinsing with PBS, the sections were mounted with fluorescence mounting medium (Dako) and viewed under the Olympus BX40 microscope with a digital microscope camera C11440 (Hamamatsu). Immunofluorescent images were systematically and randomly sampled from the primary auditory cortex (AI) area with the experimenters “blind” to the sample groups. All the images for the same experiment were taken on the same day using the same parameters. The Paxino’s Mouse Brain Atlas was used to determine the location of AI. Analysis of the IBA-1 staining was also performed with the software ImageJ. Each microglial cell and its soma were outlined by an experimenter who was “blind” to the sample groups (Fig. 3B). The image was then thresholded in ImageJ. The numbers of pixels of the whole microglial cell and its soma were counted in ImageJ, and converted to micrometers square. The soma-to-whole cell size ratio of the microglia was used to measure microglial deramification in the AI area. For PV+ and SOM+ neuron analysis, the area of AI was calculated and the numbers of PV+ neurons and SOM+ neurons were quantified in ImageJ manually. PV+ neuron density and SOM+ neuron density (numbers of cells/mm^2^) were then compared between the control and noise-exposed animals of the C57BL/6 and FVB strains.

### Real-time polymerase chain reaction

The procedure was modified from those of previous reports^22,45^. Ten days after noise exposure mice were deeply anesthetized with isoflurane and decapitated. Samples of AI tissue were collected and stored in 100 μL RIPA buffer (150 mM sodium chloride, 1.0% Triton X-100, 0.5% sodium deoxycholate, 0.1% sodium dodecyl sulphate, 50 mM Tris, pH 8.0) at − 80 °C. Tissues were homogenized in RIPA buffer by sonication. The total RNA was extracted by RNeasy Mini Kit (Qiagen, Valencia, CA). Immediately following extraction, the total RNA concentration and A260:A280 ratio of each sample were determined via NanoDrop 2000 (Thermo Scientific). The High Capacity cDNA Reverse Transcription kit (Thermo Fischer) was used to generate cDNA in a thermal cycler (ABI9700) for 2 h at 37 °C. Ten nanograms of complementary DNA (cDNA) were used in each reaction for real-time polymerase chain reaction (PCR) using the CFX96 real-time PCR system (Bio-Rad Laboratories, Hercules, CA). Threshold cycle (Ct) values of the target genes were normalized to the endogenous control gene (18S). The primer sequences are as follows: 5′-GGACTAGCCA GGAGGGAGAA-3′(forward) and 5′-GTGGTTTGCTACGACGTGGG-3′ (reverse) for TNF-α and 5′-GTAACCCGTTGAACCCCATT-3′(forward) and 5′- CCATCCAATCGGTAGTAGCG-3′ (reverse) for 18S. Differential expression between control and exposure groups was calculated using the comparative Ct method.

### Statistics

ANOVAs and post hoc analysis were done to determine significant interactions between groups or experimental procedure. Data are presented as mean ± SEM.
